# Extracellular volume fraction in dilated cardiomyopathy patients without obvious late gadolinium enhancement: comparison with healthy control subjects

**DOI:** 10.1186/1532-429X-17-S1-P288

**Published:** 2015-02-03

**Authors:** Dong Jin Im, Yoo Jin Hong, Chul Hwan Park, Byoung Wook Choi, Tae Hoon Kim

**Affiliations:** Department of Radiology, Research Institute of Radiological Science, Severance Hospital, Yonsei University College of Medicine, Seoul, Korea (the Republic of; Department of Radiology, Research Institute of Radiological Science, Gangnam severance Hospital, Yonsei University College of Medicine, Seoul, Korea (the Republic of

## Background

To evaluate whether the extra-cellular volume fraction (ECV) measured using cardiac MRI can detect myocardial tissue changes in dilated cardiomyopathy (DCM) without late gadolinium enhancement (LGE).

## Methods

Forty-one DCM patients and 10 healthy volunteers underwent pre- and post- T1 mapping using a modified Look-Locker inversion recovery (MOLLI) sequence, LGE, and cine MRI on a 3-T cardiac magnetic resonance (CMR) system. LGE-MR findings were used to divide DCM patients into two groups: Group A had no apparent LGE, and Group B had LGE apparent in at least one segment. The ECV of left ventricle (LV) myocardium (16 segments) was calculated in short-axis view as follows: ECV = [(ΔR1 of myocardium/ΔR1 of LV blood pool)] × (1 - hematocrit), where R1=1/T1, ΔR1 = Post-contrast R1 - Pre-contrast R1. The LV ejection fraction (LVEF) was obtained from cine MRI images. The mean myocardial ECV in LGE (-) segments in Group A+B was compared to that of controls. The mean myocardial ECV in Group A was compared to that of LGE (-) segments in Group B. The correlation between LV systolic function and the mean myocardial ECV of the whole myocardium was evaluated in all groups.

## Results

Among the 41 DCM patients, 22 were in Group A, and 19 were in Group B. The mean ECV of DCM patents (n = 41, 568 segments, 30.7% ± 5.9) was significantly higher (p < 0.001) than that of the control group (n = 10, 157 segments, 25.6% ± 3.2). The ECV was inversely related to LVEF in Group A (r = -0.551, p = 0.008), Group B (r = -0.525, p = 0.021), and Group A+B (r = -0.550, p < 0.001).

## Conclusions

The ECV measured by MRI could be a useful parameter in evaluating diffuse myocardial changes in DCM patients.

